# Growth gains from selective breeding in a spruce hybrid zone do not compromise local adaptation to climate

**DOI:** 10.1111/eva.12525

**Published:** 2017-09-03

**Authors:** Ian R. MacLachlan, Sam Yeaman, Sally N. Aitken

**Affiliations:** ^1^ Department of Forest and Conservation Sciences Faculty of Forestry University of British Columbia Vancouver BC Canada; ^2^ Department of Biological Sciences University of Calgary Calgary AB Canada

**Keywords:** artificial selection, assisted gene flow, climate change, *Picea engelmannii*, *Picea glauca*, tree improvement

## Abstract

Hybrid zones contain extensive standing genetic variation that facilitates rapid responses to selection. The *Picea glauca *× *Picea engelmannii* hybrid zone in western Canada is the focus of tree breeding programs that annually produce ~90 million reforestation seedlings. Understanding the direct and indirect effects of selective breeding on adaptive variation is necessary to implement assisted gene flow (AGF) polices in Alberta and British Columbia that match these seedlings with future climates. We decomposed relationships among hybrid ancestry, adaptive traits, and climate to understand the implications of selective breeding for climate adaptations and AGF strategies. The effects of selection on associations among hybrid index estimated from ~6,500 SNPs, adaptive traits, and provenance climates were assessed for ~2,400 common garden seedlings. Hybrid index differences between natural and selected seedlings within breeding zones were small in Alberta (average +2%), but larger and more variable in BC (average −7%, range −24% to +1%), slightly favoring *P. glauca* ancestry. The average height growth gain of selected seedlings over natural seedlings within breeding zones was 36% (range 12%–86%). Clines in growth with temperature‐related variables were strong, but differed little between selected and natural populations. Seedling hybrid index and growth trait associations with evapotranspiration‐related climate variables were stronger in selected than in natural seedlings, indicating possible preadaptation to drier future climates. Associations among cold hardiness, hybrid ancestry, and cold‐related climate variables dominated signals of local adaptation and were preserved in breeding populations. Strong hybrid ancestry–phenotype–climate associations suggest that AGF will be necessary to match interior spruce breeding populations with shifting future climates. The absence of antagonistic selection responses among traits and maintenance of cold adaptation in selected seedlings suggests breeding populations can be safely redeployed using AGF prescriptions similar to those of natural populations.

## 1 INTRODUCTION

Natural hybrid zones contain large amounts of standing genetic variation that provides the raw material for rapid selection responses and transgressive adaptive phenotypes, even if the selection pressure is relatively weak (Barrett & Schluter, 2008; Rieseberg, Archer, & Wayne, 1999). Where gene flow from progenitor populations is ongoing, admixture facilitates adaptive introgression among hybrids and parents (Buerkle & Lexer, 2008). Admixture can also be sufficient to generate divergent phenotypes that allow rapid ecological transitions into novel environments (Rieseberg et al., 2003). From a genetic conservation and forest management perspective, the ability for hybrids to adapt rapidly to changing environmental conditions could be useful for managing and promoting adaptation in populations that are threatened by anthropogenic environmental change (Aitken, Yeaman, Holliday, Wang, & Curtis‐McLane, 2008; Hamilton & Miller, 2016).

Hybridization as a source of genetic variation and adaptive introgression is being increasingly documented in natural populations of forest trees (Cullingham, James, Cooke, & Coltman, 2012; De Carvalho et al., 2010; Hamilton, De la Torre, & Aitken, 2015; Suarez‐Gonzalez et al., 2016). Many forest trees, particularly outcrossing wind‐pollinated species, already harbor large amounts of additive genetic variation that facilitate adaptive responses to novel environments (Kremer et al., 2012). It is a reasonable expectation that where species hybridize, adaptive responses to selection will be enhanced further, in the absence of hybrid incompatibility or outbreeding depression. Species within some of the world most important commercial timber genera readily hybridize to produce fit, fertile offspring, for example, *Eucalyptus*,* Pinus*,* Picea,* and *Populus*, and tree breeders take advantage of this. Artificially crossing parental taxa generates heterosis in some cases, and a greater range of additive genetic variation for selection and accelerated genetic gain, but F1 progeny from artificial hybrid breeding programs are usually limited to high‐gain plantation forestry systems. Alternatively, breeding programs may select on genetic variation in populations within natural hybrid zones that are adapted to local environmental conditions and are suitable for reforestation objectives that include maintaining long‐term forest productivity, health, and ecosystem function. In the western Canadian “interior spruce” hybrid zone, these objectives of increased timber yields from selective breeding and natural forest management coincide.

“Interior spruce” is used to describe hybrid populations composed of white spruce (*Picea glauca* (Monech) Voss) and Engelmann spruce (*Picea engelmannii* Parry ex Engelm.) ancestry, although some western populations also contain small amounts of ancestry from submaritime Sitka spruce (*Picea sitchensis* (Bong.) Carr.) (Hamilton et al., 2015; Roche, 1969). British Columbia (BC) was the convergence point of these three species' ranges following postglacial recolonization (Daubenmire, 1974; De La Torre, Roberts, & Aitken, 2014), but low levels of *P. glauca* ancestry are also found in *P. engelmannii‐*dominated hybrids that extend south into the USA (Haselhorst & Buerkle, 2013). In Alberta (AB), interior spruce is largely represented by pure *P. glauca* genotypes, except in the Rocky Mountains and Rocky Mountain foothills where low levels of *P. engelmannii* ancestry are present. *Picea engelmannii* is a mid‐ to high‐elevation species, but is more or less continuously distributed throughout subalpine forests in the southern half of BC between 1,000 and 2,200 m elevation (Alexander, 1987; Coates, Haeussler, Lindeburgh, Pojar, & Stock, 1994). *Picea glauca* has a continuous transcontinental, boreal, and sub‐boreal distribution at low to mid‐elevations (<1,500 m) from eastern Canada to BC and western Alaska (Nienstaedt & Zasada, 1990).

*Picea glauca *× *P. engelmannii* hybrids have formed from extensive multigenerational crossing and backcrossing, resulting in asymmetric introgression that favors *P. engelmannii* in a contact zone between these two species that may predate the last glacial maximum (De La Torre, Roberts, et al., 2014; Haselhorst & Buerkle, 2013). Adaptive ecological divergence preserves *P. glauca* and *P. engelmannii* as distinct species, but the interior spruce hybrid zone is maintained by greater fitness of hybrid individuals in intermediate local environments (De La Torre, Jaquish, & Aitken, 2014). *Picea engelmannii* is adapted to long montane winters with high snowfall, and short, cool, humid summers (Alexander, 1987). By contrast, *P*. *glauca* is adapted to extreme boreal and sub‐boreal winter cold, warm summers, and low annual precipitation (Nienstaedt & Zasada, 1990). Proportions of parental ancestry in hybrids are driven by adaptation to summer aridity and winter precipitation as snow. Hybrid fitness in intermediate habitats follows regional gradients in latitude and temperature, as well as local climatic gradients associated with elevation (De La Torre, Jaquish, et al., 2014). Across AB and BC interior spruce populations, height growth in common gardens is greatest in populations from the valleys of southern BC, and decreases as tolerance to extreme cold increases with latitude. Growth also decreases locally as tolerance of deep snow pack and short growing seasons increases with elevation (De La Torre, Jaquish, et al., 2014; Gray et al., 2016; Liepe, Hamann, Smets, Fitzpatrick, & Aitken, 2016; Rehfeldt, 1994).

Effective reforestation using interior spruce progeny from selective breeding programs requires planting genotypes that are adapted to local climates. Local adaptation to climate in interior spruce selective breeding programs has been managed in AB and BC since the 1960s using geographically and elevationally defined breeding and seedlot deployment zones. The objective of breeding in these zone‐specific programs is to increase genetic worth for timber volume, while maintaining local adaptation and genetic diversity. Interior spruce breeding programs do not have discrete generations and reflect continuous tree improvement progress; most BC programs are at stages equivalent to second or third cycles of breeding and selection, but produce seedlots from second‐generation seed orchards. AB programs are at stages equivalent to a second cycle of breeding and selection, but produce seedlots from first‐generation seed orchards. The genetic worth of a seedlot is the average genetic gain expected at the time of timber harvesting, calculated from the breeding values of all seed orchard parent trees weighted by their gametic contribution to a seedlot (Woods, Stoehr, & Webber, 1996). Orchards produce open‐pollinated seedlots with genetic worth values for growth of 10%–30% in BC (Forest Genetics Council of British Columbia 2015) and ~2.5% in AB (A. Benowicz and S. John, personal communications). The interior spruce hybrid zone and the timber harvested from it are economically important. Annually, 85 to 90 million seedlings planted in AB and BC come from spruce selective breeding programs (Alberta Environment and Sustainable Resource Development 2011; Forest Genetics Council of British Columbia 2015).

Height gains in breeding programs can be achieved by selecting parent trees that grow faster or grow for longer, but risk incurring undesirable correlated responses to artificial selection. Pleiotropy and linkage disequilibrium among causal loci could produce a shift in trait–climate relationships for adaptive traits in selectively bred reforestation seedlots, producing responses such as reduced drought tolerance, seasonally unsynchronized growth phenology, and reduced cold hardiness (Howe et al., 2003; Pita, Cañas, Soria, Ruiz, & Toval, 2005). Seedling establishment is the most vulnerable growth phase to climatic events in the life a forest stand. Reduced tolerance to climatic stress because of selective breeding for height could result in rapid and costly seedling mortality. In a previous study, we examined the effects of selective breeding on adaptive traits in lodgepole pine (*Pinus contorta* Dougl. ex Loud. var. *latifolia* Engelm.). Trade‐offs among lodgepole pine traits were negligible and climatic adaptation was maintained in selectively bred seedlings (MacLachlan, Wang, Hamann, Smets, & Aitken, 2017). Interior spruce breeding programs of AB and BC have similar procedures for testing, selection, and breeding as lodgepole pine programs, but for the current study our expectations for correlated responses to selection among traits in interior spruce are not straightforward. Among breeding zones, hybrid ancestry will vary substantially, and within breeding zones hybrid variation has the potential to affect selection responses, even though levels of variation in height growth appear to be maintained among breeding populations (Stoehr, O'Neill, Hollefreund, & Yanchuk, 2005). In southern BC, open‐pollinated wild‐stand families with greater *P. glauca* ancestry had greater breeding values for height, cold hardiness, and tolerance of low moisture conditions, and did not show height gain–cold injury trade‐offs (De La Torre, Jaquish, et al., 2014). Across BC, breeding programs generate growth gains under local climates, and although the safe climatic transfer distances of breeding populations were diminished relative to natural populations, they were still greater than permitted in current deployment zones (O'Neill, Stoehr, & Jaquish, 2014).

As climates both warm and become more variable in western Canada, genotypes in geographically defined seed deployment zones will become dissociated from their local climates and maladapted to future climates (Aitken et al., 2008; Gauthier, Bernier, Kuuluvainen, Shvidenko, & Schepaschenko, 2015). To increase the efficiency and flexibility of seedlot deployment, AB and BC are currently moving away from geographic zones and designing climate‐based seed transfer systems. These seed transfer systems use assisted gene flow (AGF) to mitigate adaptive lags and improve the future productivity of managed forests on natural landscapes that will be repeatedly harvested and restocked in the future (Gray, Gylander, Mbogga, Chen, & Hamann, 2011; O'Neill et al., 2008). The policy shift toward AGF requires a detailed knowledge of current local adaptation to climate because AGF is expected to deploy seedlots toward the margins of their climatic niches, and trade‐off early rotation risks of phenological asynchrony and cold injury against mid‐ to late‐rotation growth gains as climates become more suitable to growth (Aitken & Whitlock, 2013; MacLachlan et al., 2017). However, artificial selection has the potential to modify relationships among climatically adaptive traits, and their associations with climate, that would make AGF prescriptions determined from natural seedlots unsuitable for selected seedlots. Accurate AGF prescriptions are important for selectively bred seedlots because they represent an increasing proportion of reforestation seedlots; policies in AB and BC mandate the deployment of selectively bred seedlots on public land, when seed supplies permit, to increase future timber yields (Alberta Forest Genetic Resources Council 2015; British Columbia MFLNRO 2010).

Selective breeding and hybrid variation add extra layers of complexity to our understanding of climatic adaptation within and among interior spruce breeding populations, and the use of AGF to redeploy breeding populations to match shifting future climates. Implementing AGF requires a nuanced understanding of how selective breeding of interior spruce modifies adaptive traits, their trade‐offs with growth within and among breeding populations, and their climatic associations across a hybrid zone that contains high levels of adaptive genetic variation. We address four primary questions. (i) How does selection modify hybrid ancestry composition within and among breeding zones? (ii) What are the effects of selective breeding on adaptive phenotypic traits and the relationships among traits? (iii) How does selection change the relationships between growth gains, adaptive traits, and climate? (iv) How do changes in the hybrid index of selected seedlots modify relationships with adaptive traits, or with climate? By answering these questions, we can inform how AGF policies should redeploy selectively bred interior spruce reforestation seedlots. Our approach combines detailed phenotypic data for growth, phenology, and cold hardiness traits with hybrid index estimates from ~6,500 SNP markers, and climate estimates for 16 variables. Decomposing the phenotypic, climatic, and hybrid ancestry components of adaptive variation allows us to describe the changes that underlie differences in ancestry within and among interior spruce breeding zones, then relate these findings to future AGF needs. We achieve this by evaluating seedlings from selectively bred seedlots relative to their natural stand counterparts using a large common garden established in benign conditions. Our study compliments long‐term replicated field trials established across AB and BC.

## 2 METHODS

### 2.1 Experimental sampling and establishment

**Figure 1:**
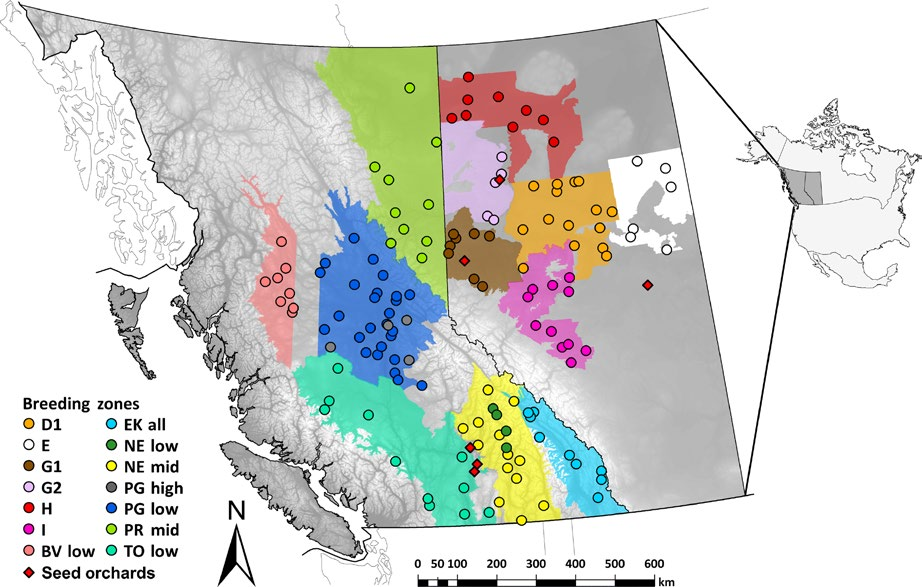
Geographic origins of the natural populations (filled circles) and selected seedling breeding zones (filled polygons) sampled from across the ranges of *Picea engelmannii*,* Picea glauca*, and their hybrid zone in AB and BC. Polygons for the NE low and PG high selected seedling breeding zones are not shown, because these two zones overlap geographically with the NE mid‐ and PG low breeding zones, respectively, but have different elevations. Diamonds indicate seed orchard locations

We sampled seedlots from 14 breeding zones defined by geography and elevation across the range of *P. engelmannii*,* P. glauca,* and their hybrids in AB and BC (Table 1). Eighteen open‐pollinated, selectively bred orchard seedlots with the highest genetic worth for growth were obtained for the most recent year available from each breeding zone (Table 1). We also obtained 138 open‐pollinated natural (wild‐stand) spruce seedlots from the same 14 breeding zones (Figure 1). Seeds from these 156 seedlots were established in a common garden on the UBC campus in Vancouver, BC, Canada. Seeds were triple‐sown at 8 cm spacing into 12 randomized incomplete blocks across two adjacent raised nursery beds filled with double‐screened sandy loam topsoil. Postgermination, seedlings were systematically thinned to leave one healthy individual, kept well‐watered, and fertilized two or three times each growing season (Peters Excel^®^ 15‐5‐15 NPK water soluble fertilizer applied at a manufacturer recommended N concentration of 200 ppm). One or two selectively bred seedlots were sampled per breeding zone, and each was represented by 66 seedlings. In each breeding zone, six randomly selected natural seedlots had 12–16 seedlings, and any additional natural seedlots had a minimum of four seedlings. A total of 2,424 (natural *n *=* *1,244, selected *n *=* *1,180) seedlings were established.

**Table 1:** Breeding zones sampled for selectively bred and natural seedlots, their elevational range, number of seedlots sampled, number of seedlings established, and for selected seedlots the number of parent tree clones in each seed orchard contributing cones to those seed lots. AB breeding zones are formally identified as D1, E, G1, G2, H, and I. BC breeding zone abbreviations are BV (Bulkley Valley), EK (East Kootenay), NE (Nelson), PG (Prince George), PR (Peace River), and TO (Thompson – Okanagan)

Province	Breeding zone	Elevation range (m)	Natural		Selected		
			Seedlots	Seedlings	Seedlots	Clones per seedlot	Seedlings
AB	D1	501–800	15	108	2	81 & 72	112
AB	E	301–650	6	72	1	87	66
AB	G1	651–1,050	8	80	1	146	66
AB	G2	501–900	5	70	1	87	66
AB	H	251–550	9	84	1	50	66
AB	I	701–1,200	11	92	1	153	66
BC	BV low	0–1,200	8	80	2	118 & 19	112
BC	EK all	750–1,700	8	88	1	26	66
BC	NE low	0–800	5	56	1	34	66
BC	NE mid	800–1,500	11	110	1	63	66
BC	PG low	600–1,200	26	152	2	19 & 53	112
BC	PG high	1,200–1,550	4	64	1	40	66
BC	PR mid	650–1,200	10	88	1	40	66
BC	TO low	700–1,300	11	92	2	30 & 41	112

### 2.2 Climatic data

To characterize climate–phenotype and climate–hybrid index relationships, we interpolated 16 climatic variables for the 1961 to 1990 climate normal period for each seedlot origin using ClimateNA version 5.21 (available at http://cfcg.forestry.ubc.ca/projects/climate-data/climatebcwna/#ClimateNA; Wang, Hamann, Spittlehouse, and Carroll (2016)). We used this climate normal period to estimate historical conditions that are likely to have shaped local adaptation of interior spruce populations, without much influence of recent climatic warming trends. The geographic variables latitude, longitude, and elevation that reflect climatic gradients were also included in our analyses (Table 2).

**Table 2:** Variation in breeding zone trait means (BLUEs) explained by breeding zone hybrid index means, for natural and selected seedlots. *p*‐values are statistically significant at an adjusted α = 0.0083 cutoff (bold font)

Traits	Natural		Selected	
	*r* ^*2*^	*p*‐value	*r* ^*2*^	*p*‐value
Height (cm)	.25	.0686	.45	.0085
Growth Rate (cm/Day)	.36	.0231	**.59**	.0014
Shoot Mass (^0.25)(g)	.36	.0241	.41	.0141
Bud Break (Day)	.27	.058	**.54**	.0027
Bud Set (Day)	.03	.5871	.12	.233
Cold Injury	**.79**	<.0001	**.83**	<.0001

The selectively bred seedlots we sampled are composed of bulked seed produced in open‐pollinated seed orchards from clones of 19–118 parent trees. Climatic variables for every parent tree contributing to a selected seedlot were estimated using ClimateNA, and averages of each parent tree's climate variables were weighted by their estimated maternal contribution to the seedlot. Maternal contributions are defined as the proportion of cones contributed to the seedlot by each seed orchard clone. This gave us the most representative climate estimate possible for each selected seedlot, because paternal contributions were inconsistently available among seed orchards.

Every seedling from the same seedlot was assigned the same provenance climatic data. Principal components analysis scores were also used to summarize each seedlot's climatic variables and included as additional climatic variables. Finally, for each climatic variable, the mean of all natural or selectively bred seedlings within a given breeding zone was calculated. All analyses were performed in R (R Core Team 2016) unless otherwise stated.

### 2.3 DNA extraction and SNP genotyping

We collected newly emerged needle tissue from each seedling in spring 2013 and DNA was extracted using a Macherey‐Nagel Nucleospin 96 Plant II Core™ kit, automated on an Eppendorf EpMotion 5075™ liquid handling platform. To genotype these samples, we used the AdapTree interior spruce Affymetrix Axiom 50K interior spruce SNP array (S.Y. Yeaman, K.A. Hodgins & S.N. Aitken, unpublished). A more detailed description of this array's design is forthcoming, but briefly, it was constructed as follows. SNP discovery was conducted using the sequence capture dataset for interior spruce described in Yeaman et al. (2016) and Suren et al. (2016), which included successful probes for the exons of >23,000 genes and a large number of intergenic regions. Intergenic regions were identified by using GMAP (Wu & Watanabe, 2005) to map the draft transcriptome for interior spruce (Yeaman et al., 2014) to the white spruce draft genome (Birol et al., 2013), with intergenic regions identified as any genomic contigs that did not have a good hit to a gene based on this mapping. The entire array design included 51,029 SNPs, of which 5144 were designed based on SNPs from intergenic regions. Intergenic SNPs were chosen for inclusion by randomly picking one diallelic SNP per identified intergenic genomic contig, and using the Affymetrix design algorithm recommendations to prioritize SNPs with a rating of either “recommended” (preferred) or “neutral” (when the contig contained no “recommended” SNPs). The Affymetrix design algorithm attempts to minimize the inclusion of regions that are repetitive in the genome or that have SNPs in the regions flanking the target SNP. Samples were genotyped using the Affymetrix array by Neogen GeneSeek (Lincoln, Nebraska).

### 2.4 Hybrid index analyses

After genotyping 2,424 individuals and filtering out SNPs that had GeneSeek quality values >0.15 (0 = high quality, 1 = low quality), and call rates <0.85, 6,482 SNPs called in 2,072 genotypes remained for hybrid index estimation. This represents a large reduction from the initial 51,029 SNPs because SNPs on the array were selected based on *P. glauca* and *P. engelmannii*, resulting in relatively low SNP quality scores and call rates for *P. sitchensis* reference genotypes.

Previously, Hamilton et al. (2015) identified a small number of natural hybrids with shared *P. engelmannii*,* P. glauca,* and *P. sitchensis* ancestry within the Bulkley Valley and Prince George breeding zones. The majority of the ancestry in these zones originated from *P. engelmannii* and *P. glauca*, with *P. sitchensis* as a minor component (~7% in Prince George). To accommodate this unbalanced hybrid ancestry, we estimated hybrid indices for each individual seedling's genotype using the projection analysis function in the software ADMIXTURE (Alexander & Novembre, 2009). Our ADMIXTURE projection analysis reference panel consisted of 500 genotypes. From the initial sample of 2,424 individuals, 45 *P. engelmannii* genotypes were selected from higher‐elevation (1,350–2,000 m) populations in south‐central BC that had >90% *P. engelmannii* ancestry (J. Degner, unpublished data, University of British Columbia, Centre for Forest Conservation Genetics), and 31 pure *P. glauca* genotypes were selected from our populations in northeastern Alberta. We included 31 additional pure *P. sitchensis* from southern Alaska (J. Elleouet, unpublished data, University of British Columbia, Centre for Forest Conservation Genetics) using the same SNP array. The remaining 393 reference panel genotypes were sampled randomly from the 2,348 individuals not already sampled for the reference panel. Hybrid ancestry of the reference panel was estimated in ADMIXTURE using *K* = 3 species (Hamilton et al., 2015), and then hybrid ancestry estimates for the remaining 1,572 genotypes were projected from population allele frequencies of the reference panel. Hybrid ancestry proportions of the reference panel were validated using *Structure* analysis (Pritchard, Stephens, & Donnelly, 2000) of 817 putatively neutral SNPs (J. Yoder, unpublished data, University of British Columbia, Centre for Forest Conservation Genetics) that were not adaptive candidates in the analyses of Yeaman et al. (2016). Unbalanced ancestry proportions from the three parental taxa meant the full sample of 2,424 individuals could not be validated in this way.

ADMIXTURE Q‐values of *P. engelmannii*,* P. glauca,* and *P. sitchensis* ancestry for each genotype were averaged for each breeding zone by seedling type combination (natural or selected), in line with the phenotypic and climatic data. Because mean *P. sitchensis* ancestry proportions in most zones were minimal, we used the proportion of *P. engelmannii* ancestry to characterize hybrid index for the purpose of comparing seedling types: Values of zero indicate complete *P. glauca* ancestry and values of one indicate complete *P. engelmannii* ancestry. Pairwise significant differences in hybrid index between seedling types within breeding zones were preceded by tests for normal distributions of each seedling type's hybrid index, and equal variances between seedling types. Equation (1) was used to estimate the amount of phenotypic variance explained by hybrid ancestry among breeding zones. Best linear unbiased estimates (BLUEs) of each breeding zone by seedling type combination trait mean (calculated from Equation (2)) were the dependent variables, mean breeding zone hybrid index was the independent variable, and seedling type was included as a categorical variable.

(1) $$y_{ij}=\beta_{0}+\beta_{1}\left(x_{1}\right)+\beta_{2}\left(x_{2}\right)+\beta_{3}\left(x_{1}x_{2}\right)+e_{ij}$$

where *y*_*ij*_ is the BLUE of seedling type *i* in breeding zone *j*;* x*_1_ is the hybrid index; *x*_2_ is the categorical covariate “seedling type”; β_0_ is the intercept; β_1_ and β_2_ are the hybrid index and seedling type coefficients, respectively; β_3_ is the coefficient of the seedling type by hybrid index interaction; and *e*_*ij*_ is the residual error of *y*_*ij*_.

### 2.5 Phenotypic data

Seedlings were phenotyped for six growth, phenology, and cold injury traits during the second (2013) or third (2014) growing seasons. Seedling height (cm) and shoot dry mass (g) were measured during season three. From 14 season two height measurements, the growth curve analysis described in MacLachlan et al. (2017) was used to estimate growth rate (cm day^−1^) as the tangent of a four‐parameter sigmoid growth curve. Bud break was recorded weekly from late March to late May of the second growing season, and classified as visible needle emergence from within the apical bud scales. Bud set was recorded weekly from mid‐June to mid‐September of the second growing season, and defined as the presence of brown apical bud scales. Timing of bud break and that of bud set were translated to day of year starting January 1st.

Autumn cold injury was determined from electrolyte leakage testing of needles following the artificial freeze testing protocol described in MacLachlan et al. (2017). Cold injury testing was completed over a 3‐week period, commencing on September 16, 2014 (season three), using −15°C and −22°C test temperatures. Flint, Boyce, and Beattie's (1967) index of cold injury (*I*) was calculated for individual seedlings at each test temperature, relative to the unfrozen control samples. Individual seedling's *I* values from both test temperatures were then averaged and mean *I* values used for our analyses. *I* represents the percent cold injury incurred; zero = undamaged; 100 = maximum freezing damage.

### 2.6 Phenotypic data analyses

Breeding zone by seedling type means were estimated as BLUEs of the fixed effects using the linear mixed model in Equation (2) (MacLachlan et al., 2017) implemented in the software package ASReml‐R version 3.0 (Butler, 2009).

(2) $$Y_{ijklm}=\;\mu +\;S_{j}+Z_{k}+{\left(S{}^{*}Z\right)}_{jk}+B_{l}+L{\left(B\right)}_{lm}+e_{ijklm},$$

where $Y_{ijklm}$ is the phenotypic observation of a trait made on individual *i* from the *j*th seedling type (*S*) and *k*th breeding zone (*Z*), grown in the *l*th block (*B*), at the *m*th seedling location (*L*) nested within block ($L{\left(B\right)}_{lm}$)). S*Z is the seedling type by breeding zone interaction. μ is the experimental mean, and *e* is the residual error of individual *i*. Seedling type (natural or selectively bred) and breeding zone were fixed effects; block and location within block were random effects. Significant pairwise differences between BLUEs of seedling type means within breeding zones were tested using two‐sample *t*‐tests. Using the breeding zone by seedling type means, we then calculated pairwise trait–trait correlations for each seedling type to identify possible trade‐offs among adaptive traits resulting from selection for growth. Significant differences between the equivalent natural and selected seedling trait–trait correlation were tested as z‐scores using the *paired.r* function of the *psych* package in R.

Phenotypic clines associated with 16 climatic variables (Table 2), as well as climate PC1 and PC2 scores, were estimated for each trait using Equation (1). *X*_1_ in Equation (1) was replaced by the mean breeding zone values of a continuously distributed climate variable, and β_1_ was the corresponding climatic variable's regression coefficient.

### 2.7 Climatic biases

Within each breeding zone, we calculated the difference between natural seedling mean annual temperature (MAT) and the mean MAT of selected seedlings weighted by maternal contribution to seedlots. Similarly, we calculated the percent height gain from natural to selected seedlings in each zone. Height gains were regressed on MAT differences to test the hypothesis that growth gains occur because parent trees in breeding programs tend to be sourced from warmer, more productive sites within breeding zones.

The embryos of interior spruce seeds develop between July and late August (Owens & Molder, 1984). We tested for epigenetic effects on seedlings traits due to seed orchard temperature during embryo development. Differences were calculated between the mean summer temperatures (MST) (June to August) of breeding zones and their respective seed orchard locations in the years our seedlots were produced. Height gains among breeding zones were regressed on these MST differences.

Our common garden test site is located on a mild, moist coastal site, outside the natural range of interior spruce. To test effects of common garden location on seedling growth, we calculated the difference between each breeding zone's mean MAT of selected seedlings and the test site MAT, then regressed seedling height gains upon these MAT differences.

## 3 RESULTS

Ninety‐two percentage of seedlings germinated and survived for three growing seasons without incurring damage that compromised their genotypic or phenotypic data. Of these, individual height growth curves were successfully modeled for 2,058 of 2,249 seedlings. Average natural and selected growth curves had *R* ^2^ estimates of 0.879 and 0.904, respectively.

### 3.1 Hybrid index means

Estimates of hybrid index from ADMIXTURE for the projection analysis reference population were strongly correlated with *Structure* hybrid index estimates for the same samples (*r* = .99, *p* < .0001) (Fig. S1). Interior spruce populations from AB were dominated by *P. glauca*, where the maximum hybrid index (proportion of *P. engelmannii* ancestry) of natural seedlings was 0.10 in breeding zone G1, immediately adjacent to the central AB–BC border. In BC, hybrid index of natural seedlings varied among breeding zones from 0.19 (PR mid) to 0.89 (NE mid), averaging 0.53 (Table S1). The difference in hybrid index between natural and selected seedlings in AB breeding zones was 2% or less, and so here we focus only on BC breeding zones.

**Figure 2:**
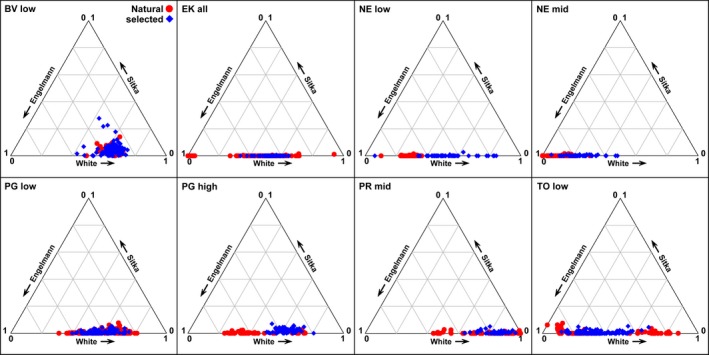
Ternary plots representing the proportion of Engelmann (*Picea engelmannii*), white (*Picea glauca*), and Sitka (*Picea sitchensis*) spruce ancestry of individual seedlings within the BC breeding zones. All AB breeding zones have >90% white spruce ancestry and are omitted from this figure

The effects of selective breeding on hybrid ancestry varied substantially among BC breeding zones. Ternary plots show several trends within breeding zones (Figure 2). (i) The majority of interior spruce ancestry in BC derives from *P. engelmannii* and *P. glauca*. Ancestry from *P. sitchensis* is most prominent in BV low, but the *P. sitchensis* components of hybrid indices were low and similar between seedling types (both ~4%), except for a few outliers. Elsewhere, average *P. sitchensis* contributions to ancestry are negligible (≤0.02%). (ii) For breeding zones with primarily *P. engelmannii *× *P. glauca* ancestry, variation in ancestry among trees within zones is less in selected seedlings than in natural seedlings, with the exception of NE low. (iii) Selection and breeding have shifted the distribution of ancestry proportions toward *P. glauca* in several breeding zones including NE low, NE mid, PG high, and PR mid. The greatest difference between seedling types within a zone was in NE low, where hybrid index was 24% lower in selected than in natural seedlings, while hybrid indices of selected seedlings in NE mid and PG high were 18% and 14% lower, respectively (Table S1, Fig. S2). In contrast, PG low and TO low both had hybrid index values that were 4% greater in selected than in natural seedlings. We did not test the significance of differences between seedling types, because within all breeding zones the distributions of natural seedling hybrid index values were disparate, inconsistent and would invalidate assumptions of equal variances for pairwise statistical tests. However, small standard errors of hybrid index means (Table S1, Fig. S2) suggest these differences between seedlot types are substantive.

### 3.2 Hybrid index–trait relationships

**Figure 3:**
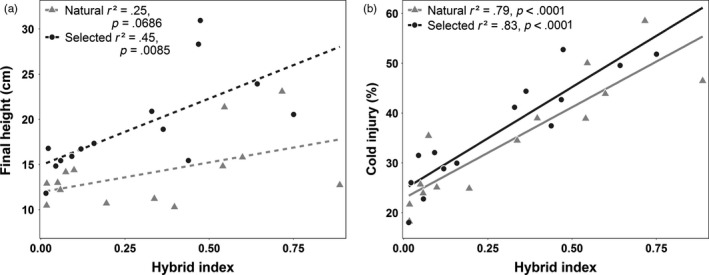
Regressions of (a) height and (b) cold injury versus hybrid index. Points represent BLUEs of trait means (Table S4, Figure 5), and the mean spruce hybrid index (*Picea engelmannii* proportion) for each breeding zone by seedling type combination (Table S1). *p*‐values are statistically significant at the adjusted α = 0.0083 cutoff used in Table 3

For all traits, hybrid index explained more of the phenotypic variance in selected than in natural seedlings, although differences in *r* ^2^ values between seedling types varied greatly among traits (Table 3). In general, growth trait–hybrid index relationships were weaker than growth trait–climate relationships (Table S5). Hybrid index did not explain significant variation among breeding zones in final seedling height (Figure 3a), but growth rate in selected seedlings was significantly related to hybrid index (*r* ^2^ = .59). Bud break timing had a significant relationship with hybrid index only in selected seedlings (*r* ^2^ = .54), while bud set timing had no relationship with hybrid index for either seedlot type (Table 3). In contrast, cold injury–hybrid index relationships were strong and statistically significant, but differed very little between seedling types (natural *r* ^2^ = .79, selected *r* ^2^ = .83) (Figure 3b), reflecting the strong clines in cold injury with low temperature‐related climatic variables (Table S5).

**Table 3:** Variation in breeding zone hybrid index means explained by breeding zone climate means for natural and selected seedlots. *p*‐values are statistically significant at an adjusted α = 0.0028 cutoff (bold font)

Geographic or Climatic Variables	Acronym	Natural		Selected	
		*r* ^*2*^	*p*‐value	*r* ^*2*^	*p*‐value
Latitude (^o^N)	LAT	**.75**	<.0001	**.73**	<.0001
Longitude (^o^W)	LONG	.13	.2141	.05	.4617
Elevation (m)	ELEV	.41	.0131	.35	.027
Mean annual temperature (^o^C)	MAT	**.68**	.0003	**.62**	.0008
Mean warmest month temperature (^o^C)	MWMT	.02	.6104	.00	.9782
Mean coldest month temperature (^o^C)	MCMT	**.75**	<.0001	**.75**	<.0001
Log mean annual precipitation (mm)	log MAP	**.76**	<.0001	.34	.0284
Mean annual summer precipitation (mm)	MSP	.03	.5421	.23	.0816
Summer heat moisture index	SHM	.03	.5558	.26	.063
Degree days below zero (^o^C)	DD < 0	**.74**	<.0001	**.71**	.0002
Growing degree days above five degrees (^o^C)	DD > 5	.01	.8154	.04	.4757
End of frost‐free period (day)	eFFP	**.58**	.0017	.48	.0062
Precipitation as snow (mm)	PAS	**.84**	<.0001	.47	.0071
Extreme minimum temperature (^o^C)	EMT	**.88**	<.0001	**.83**	<.0001
Extreme maximum temperature (^o^C)	EXT	.00	.9591	.09	.3132
Hargreaves climatic moisture deficit (mm)	CMD	.10	.2737	.38	.0188
Climatic principle component 1 scores	PC1	.51	.0039	**.54**	.0027
Climatic principle component 2 scores	PC2	.52	.0038	.32	.0347

### 3.3 Hybrid index clines

**Figure 4:**
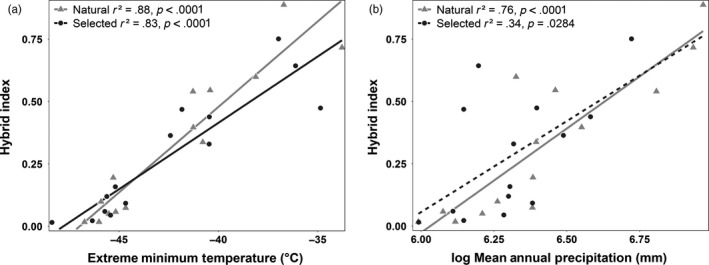
Examples of climatic clines in hybrid index. EMT (a) has the strongest climatic cline, while log MAP (b) has the cline in hybrid index with the greatest difference between seedling types. Points represent mean hybrid index (proportion of *Picea engelmannii* ancestry, Table S1) and mean climatic values for each breeding zone by seedling type combination. *p*‐values are statistically significant at the same adjusted α = 0.0028 cutoff used in Table 2 for 18 climatic comparisons per trait and seedling type

Large amounts of variation in interior spruce hybrid index were explained by latitude, mean annual temperature, and cold‐related variables (mean coldest month temperature, degree days below zero, and extreme minimum temperature) (Table 2). The most striking result from clines in hybrid index along these climatic gradients is their very similar *r* ^2^ values between seedlot types (Δ*r* ^2^ ≤ .05), indicating that selective breeding is not significantly altering these relationships (e.g., Figure 4a). However, the clines in hybrid index with log mean annual precipitation and precipitation as snow show substantial differences between seedlot types with *r* ^2^ estimates for hybrid index of natural seedlings twice as large as those for selected seedlings (Table 2, Figure 4b). Conversely, mean summer precipitation, summer heat moisture index, and climatic moisture deficit explain a somewhat greater proportion of variation in hybrid index for selected seedlings (Δ*r* ^2^ = 0.2 to 0.28), but these clines were not significant (Table 2).

### 3.4 Breeding zone by seedlot type means

Of the six phenotypic traits analyzed, four had normal distributions and met homogeneity of variance assumptions without transformation. Shoot mass values were quarter‐root‐transformed and subsequently met normal distribution and homogeneity of variance assumptions. Bud set had a bimodal frequency distribution and was not transformed.

**Figure 5:**
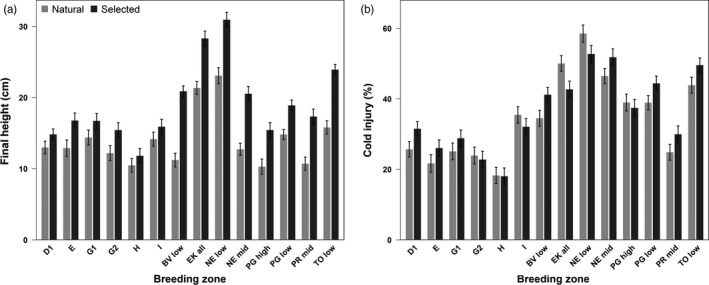
Bar plots of breeding zone‐level trait means (BLUEs) including standard error bars, for seedling (a) height and (b) cold injury

Mean seedling height was greater for selected than for natural seedlings in all breeding zones (Table S4, Figure 5a), and significantly greater in 10 of 14 zones. Height gains in selected seedlings from BC breeding zones ranged from 28% to 86% (average 51%), while AB breeding zones had more modest gains ranging from 12% to 30% (average 19%). In 11 of 14 breeding zones, growth rate per day was greater in selected seedlings, and significantly so in seven zones (Fig. S3a). Selected seedlings had greater shoot dry mass in all breeding zones, and differences between seedlot types were statistically significant in 12 of 14 zones (Fig. S3b).

Selective breeding had small effects on mean bud break timing. Mean bud break varied by 14 days across all breeding zones and seedlot types, and differed between seedlot types within zones by a maximum of 5.1 days (Table S4, Fig. S3c). In AB, the direction of bud break differences between seedlot types was inconsistent, but in BC, selected seedlings broke bud 0.5 to 5 days earlier (average 1.5 days) in every BC breeding zone except NE low (2.5 days later). Even so, significantly earlier bud break only occurred in selected compared to natural seedlings from PG low and TO low.

Bud set timing showed far greater variation among breeding zones than bud break timing, with mean bud set date varying 55 days across all breeding zone by seedling type combinations. Bud set was delayed from 3.5 to 29 days (average 13 days) in selected seedlings across all breeding zones, significantly so in nine zones (Table S4, Fig. S3d), and generally delays associated with selection were greater in BC than in AB. BV low and PR mid had the greatest difference in bud set timing between selected and natural seedlings; bud set was 29 days later in selected seedlings in both zones.

Cold injury exhibited no consistent differences between natural and selected seedlings among breeding zones (Table S4, Figure 5b), despite the generally delayed bud set timing of selected seedlings. The range of average cold injury differences between seedling types within breeding zones was −7% to +7%. Mean cold injury of selected seedlings was less than that of natural seedlings in the EK and NE low breeding zones, even though relatively large height gains were observed in these zones. By contrast, BV low had the greatest increase in cold injury associated with selection (7%), as well as the greatest relative (86%) and absolute height gains of any zone compared to the respective natural seedling mean.

### 3.5 Trait–trait correlations

Correlations among growth traits, as well as between growth traits and bud break, bud set, and cold injury, were uniformly strong (*r* ≥ .73) and individually significantly different from zero (α = 0.0033) (Table S6). Selected seedlings tended to have slightly stronger correlations among traits than natural seedlings, although differences were small (maximum Δ*r* = .14) with the exception of growth rate versus bud set. The correlation coefficient between bud break and bud set decreased slightly from natural to selected seedlings (Δ*r* = .18), while it increased slightly between bud set and cold injury (Δ*r* = .17). None of the differences between natural and selected seedlings for any trait–trait correlation were statistically significant.

### 3.6 Phenotypic clines

The first principal component of climate variables explained 41% of climatic variation, while PCs 2, 3, and 4 cumulatively accounted for 72%, 87%, and 93% of the total climatic variation (Table S2). PC1 loadings were dominated by low and average temperature variables, while PC2 loadings were harder to summarize (Table S3). Breeding zone means of PC1 and PC2 scores were used in clinal analyses to summarize phenotypic variation associated with climate in addition to the individual climate variables.

After Bonferroni adjustment for multiple comparisons within each seedling type using an α = 0.0031 cutoff value, most traits showed significant clinal variation along gradients of latitude, mean annual temperature, mean coldest month temperature, degree days below zero, extreme minimum temperature (EMT), and climate PC1 (Table S5). In most cases, these variables explained greater proportions of variation in selected than in natural seedlings. For growth traits, the derived variables climatic moisture deficit and summer heat moisture index integrating temperature and precipitation also explain a greater proportion of variation in selected seedlings. Clines with longitude, elevation, extreme maximum temperature, precipitation variables, and climatic PC2 were mostly moderate to weak and not statistically significant (except in one case, cold injury of natural seedlings versus log mean annual precipitation). Phenotypic trait means consistently had strong relationships with mean annual temperature (MAT), and so we use them here to illustrate clinal differences between natural and selected seedlings.

**Figure 6:**
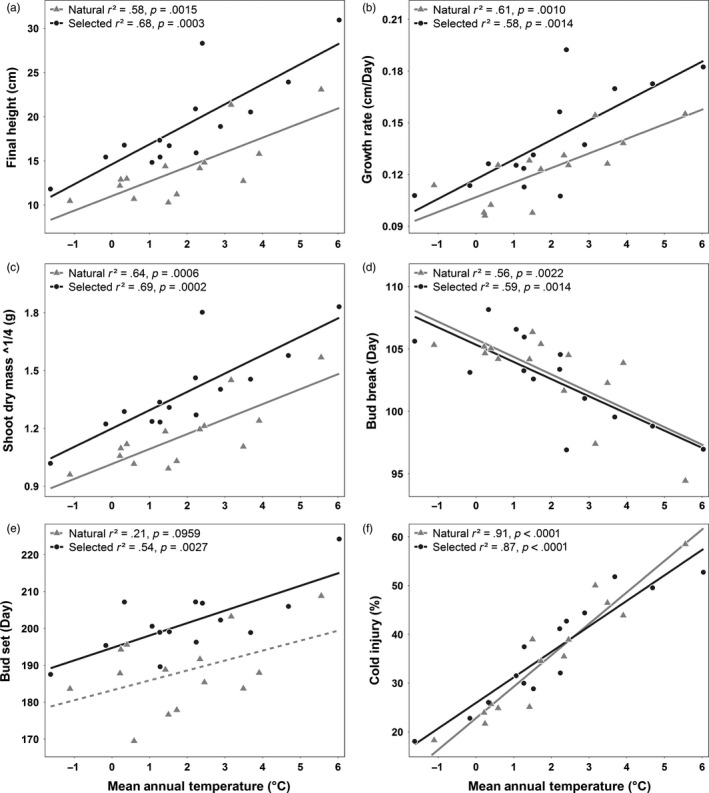
Clines in MAT for (a) height, (b) growth rate, (c) shoot dry mass, (d) bud break, (e) bud set, and (f) cold injury. None of the interactions between seed lot types are significant. Points represent trait means (BLUEs, Table S4), and climatic means for each of 14 breeding zones. *p*‐values are statistically significant at the same adjusted α = 0.0028 cutoff used in Table S5 for 18 climatic comparisons per trait and seedling type

Clines in the three growth traits associated with MAT were moderate to strong, and individually significant, but all had nonsignificant differences in cline slope between seedling types (Figure 6a–c). Bud break has a moderate to strong relationship with MAT (*r* ^2^ = ~0.6), but cline slopes did not differ significantly between seedling types (Figure 6d). Bud set only varied significantly with MAT for selected seedlings (*r* ^2^ = 0.54) (Figure 6e), and cline slopes were not significantly different between seedling types. These clines in phenology also show that seedlings from warmer breeding zones both break bud earlier and set bud later; thus, growth duration is extended at both ends of the growing season compared to colder breeding zones. Growth duration varied among zones from ~83 to ~118 days. Cold injury had the strongest clines in MAT of any trait, but these clines differed very little between natural and selected seedlots (Figure 6f).

### 3.7 Climatic biases

Within breeding zones, the average MATs of selected seedlings differed from natural seedlings by −0.76°C to +0.78°C (average 0.14°C), and among breeding zones, height gains were weakly associated with MAT differences between seedling types (*r* ^2^ = .14, *p* = .173). On average, mean summer temperature (MST) was greater in seed orchards than in breeding zones by 3.2°C (range +0.51 to +7.02°C), but this varied provincially. Seed orchard MSTs were greater by an average of 1.15°C (range +0.51°C to +1.5^o^c) in AB and 4.7°C (range 1.93°C to 7.02°C) in BC. Seed orchard MST differences among breeding zones had a modest significant relationship with height gains (*r* ^2^ = .41, *p* = .014) (Fig. S5). Differences between mean breeding zone MATs for selected seedlings and our Vancouver test site MAT were large (average 9.3°C, range 5.2°C to 12.8°C), but among breeding zones these differences did not explain variation in height gains (*r* ^2^ = .15, *p* = .173).

## 4 DISCUSSION

Hybrid ancestry and the corresponding adaptive phenotypes of selectively bred interior spruce seedlings appear to have been refined for increased growth within the constraints of local breeding zone climates. Growth gains result from both increased growth rate and delayed bud set of selected seedlings, but have not compromised the adaptive synchrony of autumn cold hardiness development relative to their natural counterparts. *P. glauca–P. engelmannii* hybrid index has weak to moderate relationships with growth traits and strong relationships with cold injury. Cold temperature‐related climate variables explain the greatest proportions of variance among populations in both hybrid index and phenotypic traits, although the strength of relationships among hybrid index, cold injury, and cold temperature‐related climate variables differ little between natural and selected seedling types. This suggests that cold hardiness is closely tied to hybrid ancestry and not compromised by selective breeding. Strong relationships among hybrid index, phenotypic traits, and climate among breeding zones mean that AGF reforestation prescriptions will become increasingly necessary to match the adaptive profile of selectively bred interior spruce seedlots to current and future climates. The same prescriptions can be used for both natural and selectively bred seedlots because there appear to be no major costs of increased growth to adaptive phenology and cold hardiness.

### 4.1 Selective breeding and hybrid ancestry

Coarse‐scale patterns of hybrid ancestry reflect large differences in the extent of hybridization between AB and BC. AB is dominated by *P. glauca* genotypes and hybrid ancestry changes from selective breeding are small (Fig. S2), implying either *P. engelmannii* alleles are maladaptive in those environments and do not offer growth gains in unsuitably cold, dry climates, or adaptive alleles are at too low a frequency to respond strongly to selection. In contrast, hybrid ancestry of natural populations is heterogeneous in BC, spanning the range of pure *P. engelmannii* to pure *P. glauca* genotypes.

Changes in the distribution of individual seedling and average hybrid index values and within BC breeding zones (Figure 2, Table S1) indicate the effects of selective breeding on hybrid ancestry composition vary among breeding zones. We observe shifts in hybrid index means toward *P. glauca* parental genotypes in selected seedlings from EK and NE breeding programs of southeastern BC that are consistent with De La Torre, Jaquish, et al. (2014). Increased *P. glauca* ancestry in the EK breeding population reflects selection on standing genetic variation within this breeding zone. However, the NE low and NE mid‐breeding populations each contain 10 pure *P. glauca* genotypes sourced from Ontario in Eastern Canada and tested for growth under local conditions in BC. In NE low and NE mid, these parent trees provided ~16% and ~6% of the respective maternal contributions to seedlots that we sampled based on cone production, and probably underlie substantial shifts toward *P. glauca* ancestry in these naturally *P. engelmannii*‐dominated zones. Within those BC breeding populations that were selected entirely from local standing genetic variation (all zones except NE), decreased variation in seedling hybrid index values (Figures 2 and S2) suggests that selection for growth is favoring a narrower range of genotypes.

Across breeding zones, hybrid index explains low to moderate proportions of variation in the growth traits of natural seedlings (Table 3). These relationships are much stronger than the similar latitude versus growth comparisons of Roche (1969), whose *P. glauca *× *engelmannii* hybrid index estimated from cone morphology broadly corresponded to latitude. Hybrid index explains a greater proportion of variation in the growth traits of selected seedlings, and slope differences between seedling types appear to be driven by a combination of hybrid index changes favoring *P. glauca* and height gains that are independent of hybrid index shifts in BC breeding zones (Figure 3a). By contrast, cold injury in artificial freezing tests is closely associated with ancestry. Seedlings with greater *P. engelmannii* ancestry suffered greater cold injury, but the differences between seedling types are negligible (Figure 3b). Strong clines in hybrid index with cold‐related climatic variables suggest low temperatures are the strongest climatic selective agents on seedlings, but again, negligible differences occur between seedling types. These relationships with hybrid index reflect both the importance of cold hardiness to adaptation in the parental species, and the strongly conserved nature of cold adaptation within breeding populations.

Like Hamilton et al. (2015), we also find hybrid index clines associated with log mean annual precipitation (log MAP) and precipitation as snow (PAS), indicating these are important adaptive gradients in natural populations, but these relationships with precipitation are weaker, although not significantly different, in selected seedlings (Figure 4b). Conversely, the slightly stronger relationships of hybrid index with climatic moisture deficit (CMD) and summer heat moisture index (SHM) for selected versus natural seedlings correspond to a shift toward *P. glauca* ancestry and potentially greater tolerance for low moisture conditions over *P. engelmannii* (Table 2).

### 4.2 Effects of selection on adaptive traits

Differences between natural and selected seedlings (Figure 5a) correspond to genetic gains for growth achieved by selective breeding programs. The modest height gains in AB compared with larger gains in BC reflect greater selection intensities and an additional breeding cycle in older BC breeding programs. Large height gains are also feasible because within BC breeding populations, large pools of standing hybrid genetic variation provide the potential for rapid responses to divergent selection (Rieseberg et al., 2003). Height gains are not related to microgeographic variation in mean annual temperature (MAT) within breeding zones, or to differences between breeding zones and test site MAT. We did, however, find a modest relationship between height gains and the differences between breeding zone and seed orchard mean summer temperatures during embryo maturation, which is largely driven by height gains in BC breeding zones (Fig. S5). This relationship could be an epigenetic consequence of warmer than native seed maturation environments of seed orchards (Bräutigam et al., 2013), but we suspect this effect is weak or nonexistent because height gains among breeding zones reflect the realized gains of breeding programs determined from long‐term trials. We cannot test these effects directly with these data. Seed orchards are located at opposite ends of the temperature gradient that our populations sample (Figure 1), with low genetic gain Alberta seed orchards located in north‐central Alberta and the high‐gain BC orchards mostly in the warm Okanagan Valley of southern BC. It is impossible to separate genetic from potential epigenetic effects because genetic gain and seed orchard climate are correlated.

The effects of selection on cold injury within breeding zones were small (Figure 5b). Some breeding zones had increases in height associated with slight increases in cold injury, but selected seedlings from southern BC breeding zones had both increased height and decreased cold injury, corresponding with shifts toward fast‐growing, cold‐resistant *P. glauca* ancestry, consistent with De La Torre, Jaquish, et al. (2014). from this region of the hybrid zone. Even so, changes in ancestry do not necessarily underlie phenotypic responses to selection. For example, while BV low has the greatest height gains of any breeding zone and slightly greater cold injury in selected seedlings, average ancestry proportions in BV low remained identical (Δ~1%) between seedling types (Figures 2 and S2). Selection does not appear to have favored introgression from faster‐growing trees with more *P. sitchensis* ancestry in this breeding zone (Hamilton et al., 2015).

Pairwise correlations among traits were uniformly strong, but differed little between seedling types (Δ*r* ≤ .17) for 13 of 15 pairwise trait combinations (Table S6). This suggests any negative trade‐offs among height growth, adaptive phenology, and cold injury that might constrain gains are weak within breeding populations. Even though bud set–growth rate correlations increased modestly (Δ*r* ~.3) with selection, seedlings that grew faster and set bud later had negligible increases in cold injury.

We studied growth and phenology traits that are known have adaptive associations with climate across species, with relatively strong population differentiation (*Q*_st_) (Savolainen, Pyhajarvi, & Knurr, 2007). Although these traits are likely to be composites of other adaptive traits or correlated with adaptive traits that we did not measure, in many instances they are characterized by strong associations with climate. Clines in growth traits along gradients in mean annual temperature (MAT) explained moderate to large proportions of variation among breeding zones, but differences in slopes between seedling types were small (Figure 3a–c). Greater cline intercepts but similar slopes of selected relative to natural seedlings suggest that gains in growth are relatively similar across the range of MAT variation in interior spruce. Among breeding zones, MAT reflects variation in mean climate, but does not necessarily capture the climatic extremes that cause physiological stress and drive local adaptation. CMD and SHM, as proxies for moisture stress, and the cold‐related variable EMT all have strong relationships with growth. Selected seedlings have stronger clines in growth traits with CMD and SHM than natural seedlings, corresponding to the stronger relationships we observed in selected seedlings between these two variables and hybrid index. The greater proportion of variation among breeding zones explained by CMD and SHM for selected seedling growth traits suggests that within breeding populations, hybrid seedlings may be more able to tolerate reduced local moisture conditions in the future. Growth trait clines with EMT explained 1.5 to 2 times as much variance in selected seedlings as in natural seedlings. Mean coldest month temperature shows a similar pattern, as does degree days below 0°C, although this latter variable reflects average rather than extreme cold. Bud break clines with MAT were identical between seedling types and unexpectedly strong given that Roche (1969) only found weak bud break associations with latitude across a wide range of BC interior spruce provenances, and in many other conifer species, this trait varies little among populations (Howe et al., 2003). Our result probably originates from an expanded range of standing genetic variation for bud break phenology across these three hybridizing species.

Even though selective breeding changes the strength of some pairwise climate–trait relationships, our results suggest there is little overall effect of selection on adaptive trait–climate relationships. Height gains appear to be derived from both delayed bud set and faster growth rate, although we cannot separate their specific effects. Height–growth rate correlations increased with selection by 0.14 (Table S6), while clines in selected seedling growth rate and bud set with climate PC1 were also slightly stronger (Δ*r* ^2^ = +.08 and +.17, respectively). Bud set precedes development of autumn cold hardiness, but the adaptive synchrony of these two processes appears to be temperature‐mediated and flexible in response to selection (Hamilton et al., 2016; Tanino, Kalcsits, Silim, Kendall, & Gray, 2010). This is congruent with our recent lodgepole pine findings; delayed bud set did not result in adaptive compromises to cold hardiness of selected seedlings that appeared able to acquire hardiness more rapidly than their natural equivalents (MacLachlan et al., 2017). In interior spruce, we also find that bud set–cold injury correlations are weak (Table S6), and climatic associations with bud set are weak to moderate in both seedling types, unlike those for cold injury (Table S5).

Adaptation to cold dominates signals of local adaptation in our study of interior spruce. Strong associations among hybrid index, cold injury, and cold‐related climatic variables are congruent with patterns of adaptive variation in autumn cold hardiness for widespread conifer species (Bansal, Harrington, & St. Clair, 2016; Gray et al., 2016; Hurme, Repo, Savolainen, & Pääkkönen, 1997; Liepe et al., 2016; Rehfeldt, 1994), and genotype–environment associations for adaptive markers associated with cold tolerance in interior spruce and lodgepole pine (Yeaman et al., 2016). Adequate autumn cold hardiness development prior to potentially damaging temperatures is essential to seasonal climatic synchrony, survival, and growth. The need for locally adaptive autumn cold hardiness in natural populations is strong enough to be highly conserved by both interior spruce and lodgepole pine breeding programs in western Canada (MacLachlan et al., 2017), even though growth traits and bud set phenology experience strong directional selection in selected seedlings of both species.

### 4.3 Selective breeding and AGF

Deploying the correct interior spruce genotypes on reforestation sites to match current and future rather than historic climates requires a thorough understanding of the relationships among ancestry, phenotypes, and climate that underpin adaptation in this hybrid zone (Aitken et al., 2008). Selection and breeding resulted in decreased variation in hybrid ancestry within most of the selected BC breeding zone populations. Among breeding zones, relationships of growth traits with both hybrid ancestry and climate were stronger in selected than in natural seedlings, and adaptive associations between hybrid ancestry, cold hardiness, and cold‐related climate variables were always strong. This means that relatively large proportions of adaptive variation are explained by differences among breeding zones and that growth and cold hardiness of breeding populations are closely associated with breeding zone climates.

Using ClimateNA, we estimate that MAT was 0.58°C higher across AB and BC during 2005 to 2014 than during the 1961 to 1990 reference period, and warming of at least 1.7°C is expected by the 2080's (British Columbia Ministry of Environment 2015). Our results from Figure 6a suggest that breeding populations from source climates that are 2°C warmer are also ~5 cm taller, which is equivalent to 25% of the total range in mean height growth of selected seedlings among breeding zones. This implies that breeding populations experiencing 2°C of warming during a single crop rotation could incur a substantial adaptive lag in growth. Continued local seed sourcing for reforestation will result in lower productivity than if selected genotypes are better matched to new climates. Therefore, AGF should be used for deployment of selectively bred genotypes to match current and near‐future climates and optimize tree growth on reforestation sites.

Similarly adapted conifer populations in western Canada appear to be distributed over wider geographic areas than previously thought (Liepe et al., 2016; O'Neill et al., 2014). Our findings of stronger growth–hybrid index–climate relationships in selected than in natural interior spruce seedlings correspond to O'Neill et al. (2014) who found that safe transfer distances of breeding populations exceed those of current seed deployment zones, but are somewhat shorter than transfer distances of equivalent natural stand populations. By decomposing the relationships among hybrid ancestry, phenotypic traits, and climate, it seems that negative trade‐offs between growth and the climatically adaptive traits we studied in selected seedlings are small or absent relative to their natural stand counterparts. Within each breeding zone, adaptation to local climates has been effectively maintained by breeding programs that select, breed, and test genotypes on several representative local sites, where low temperatures in particular limit the growth gains that can be achieved.

This finding in interior spruce is consistent with our previous study on lodgepole pine (MacLachlan et al., 2017). It implies that within the critical seed transfer distance (see Ukrainetz, O'Neill, & Jaquish, 2011) of breeding populations under current climates, AGF prescriptions for natural and selected seedlots do not need to differ. AB and BC should be able to implement AGF that realizes growth gains from selection within breeding zones without heightened risk of cold injury, because adaptive climatic relationships are maintained in breeding populations. Additionally, the hybrid ancestry and phenotypes of selected interior spruce seedlings may be somewhat preadapted to tolerate reduced moisture conditions as the climate changes, although this bears further testing. It is also likely that in the future, seedlots will be safely deployable over more extensive areas than are presently allowed in reforestation policies. This is partly because the spatial scale of similarly adapted populations is greater than previously thought, and partially because seed movements based on climatic distance rather than geographic distance better match genotypes with environments (Liepe et al., 2016; O'Neill et al., 2014). Furthermore, where breeding populations have increased growth and decreased risk of cold injury associated with an increased proportion of *P. glauca* ancestry, it may be safe to deploy their seedlots even more extensively to achieve future gains in timber supply.

## DATA ARCHIVING

All raw phenotypic and genomic data used in this study are available from the Dryad Digital Repository: https://doi.org/10.5061/dryad.gq8m9.
