# Supplementary material for: Growth gains from selective breeding in a spruce hybrid zone do not compromise local adaptation to climate
Source: Evol Appl. 2017 Sep 3;11(2):166–81. doi: 10.1111/eva.12525 (PMC5775489; doi:10.1111/eva.12525)
Supplement: Supplementary file 1 [file EVA-11-166-s001.pdf]

Supporting Information

Table S1. Mean proportions (ADMIXTURE Q-values) of the three parental spruce species' genetic contributions to each breeding zone and seedling type. Standard errors are in brackets.

Province	Breeding Zone	Seedlot Type	Parental Spruce Species					
			Engelmann		White		Sitka	
**AB**	**D1**	**Natural**	0.05	(0.00)	0.95	(0.00)	0.00	(0.000)
**AB**	**D1**	**Orchard**	0.04	(0.00)	0.95	(0.00)	0.00	(0.000)
**AB**	**E**	**Natural**	0.02	(0.00)	0.98	(0.00)	0.00	(0.000)
**AB**	**E**	**Orchard**	0.02	(0.00)	0.98	(0.00)	0.00	(0.000)
**AB**	**G1**	**Natural**	0.10	(0.01)	0.90	(0.01)	0.00	(0.000)
**AB**	**G1**	**Orchard**	0.12	(0.00)	0.88	(0.00)	0.00	(0.000)
**AB**	**G2**	**Natural**	0.06	(0.00)	0.94	(0.00)	0.00	(0.001)
**AB**	**G2**	**Orchard**	0.06	(0.00)	0.94	(0.00)	0.00	(0.001)
**AB**	**H**	**Natural**	0.02	(0.00)	0.98	(0.00)	0.00	(0.000)
**AB**	**H**	**Orchard**	0.02	(0.00)	0.98	(0.00)	0.00	(0.000)
**AB**	**I**	**Natural**	0.07	(0.00)	0.92	(0.00)	0.00	(0.001)
**AB**	**I**	**Orchard**	0.09	(0.00)	0.91	(0.00)	0.00	(0.000)
**BC**	**BV low**	**Natural**	0.34	(0.00)	0.62	(0.01)	0.04	(0.003)
**BC**	**BV low**	**Orchard**	0.33	(0.01)	0.62	(0.01)	0.05	(0.004)
**BC**	**EK all**	**Natural**	0.55	(0.02)	0.45	(0.02)	0.00	(0.000)
**BC**	**EK all**	**Orchard**	0.47	(0.01)	0.53	(0.01)	0.00	(0.000)
**BC**	**NE low**	**Natural**	0.72	(0.01)	0.28	(0.01)	0.00	(0.000)
**BC**	**NE low**	**Orchard**	0.47	(0.02)	0.53	(0.02)	0.00	(0.001)
**BC**	**NE mid**	**Natural**	0.89	(0.01)	0.11	(0.01)	0.00	(0.000)
**BC**	**NE mid**	**Orchard**	0.75	(0.01)	0.25	(0.01)	0.00	(0.000)
**BC**	**PG high**	**Natural**	0.54	(0.02)	0.46	(0.02)	0.00	(0.001)
**BC**	**PG high**	**Orchard**	0.36	(0.01)	0.61	(0.01)	0.02	(0.002)
**BC**	**PG low**	**Natural**	0.40	(0.01)	0.60	(0.01)	0.01	(0.001)
**BC**	**PG low**	**Orchard**	0.44	(0.01)	0.55	(0.01)	0.01	(0.001)
**BC**	**PR mid**	**Natural**	0.19	(0.02)	0.80	(0.02)	0.00	(0.001)
**BC**	**PR mid**	**Orchard**	0.16	(0.01)	0.83	(0.01)	0.01	(0.001)
**BC**	**TO low**	**Natural**	0.60	(0.03)	0.39	(0.03)	0.01	(0.002)
**BC**	**TO low**	**Orchard**	0.64	(0.01)	0.35	(0.01)	0.01	(0.001)

Table S2. Summary of PC1 – 4 effects from PCA of all 22 climate variables.

	PC1	PC2	PC3	PC4
**Standard deviation**	3.019	2.601	1.898	1.028
**Proportion of Variance**	0.414	0.307	0.164	0.048
**Cumulative Proportion**	0.414	0.722	0.886	0.934

Table S3. Ranked PC1 and PC2 loadings from the PCA of all 22 climate variables.

PC1		PC2	
**Variable**	**Loading**	**Variable**	**Loading**
MAT	0.304	ELEV	0.318
eFFP	0.286	MWMT	0.298
EMT	0.284	PAS	0.295
LAT	0.269	log MAP	0.284
DD > 5	0.247	MCMT	0.263
DD < 0	0.243	EXT	0.253
MCMT	0.232	DD < 0	0.246
EXT	0.224	LONG	0.245
CMD	0.219	DD > 5	0.232
SHM	0.195	LAT	0.172
MWMT	0.187	EMT	0.168
MSP	0.105	MAT	0.130
log MAP	0.074	SHM	0.121
LONG	0.048	CMD	0.080
ELEV	0.038	eFFP	0.052
PAS	0.032	MSP	0.027

Table S4. BLUEs of trait means for each breeding zone and seedlot type combination. Standard errors are in brackets.

Province	Breeding Zone	Seedlot Type	Height (cm)		Growth Rate (cm/Day)		Shoot Dry Mass (g)		Bud Break (Day)		Budset (Day)		Cold Injury (%)	
**AB**	**D1**	**Natural**	12.99	(0.88)	0.1	(0.008)	2.24	(0.76)	105	(1.1)	195.6	(3.0)	25.7	(2.2)
**AB**	**D1**	**Orchard**	14.82	(0.75)	0.13	(0.007)	3.28	(0.65)	106.6	(0.9)	200.6	(2.6)	31.5	(2.1)
**AB**	**E**	**Natural**	12.89	(1.15)	0.1	(0.011)	2.49	(1.01)	104.7	(1.4)	194.3	(3.9)	21.7	(2.5)
**AB**	**E**	**Orchard**	16.77	(1.04)	0.13	(0.009)	3.83	(0.90)	108.2	(1.3)	207.1	(3.5)	26	(2.4)
**AB**	**G1**	**Natural**	14.39	(1.06)	0.13	(0.01)	2.95	(0.92)	104.2	(1.3)	188.8	(3.6)	25.1	(2.4)
**AB**	**G1**	**Orchard**	16.72	(1.04)	0.13	(0.01)	4.03	(0.9)	102.6	(1.3)	199.1	(3.5)	28.8	(2.4)
**AB**	**G2**	**Natural**	12.19	(1.07)	0.1	(0.01)	1.86	(0.93)	105.2	(1.3)	187.8	(3.6)	23.9	(2.4)
**AB**	**G2**	**Orchard**	15.43	(1.04)	0.11	(0.009)	2.97	(0.90)	103.1	(1.3)	195.4	(3.5)	22.8	(2.4)
**AB**	**H**	**Natural**	10.47	(0.97)	0.11	(0.01)	1.53	(0.84)	105.3	(1.2)	183.6	(3.3)	18.3	(2.3)
**AB**	**H**	**Orchard**	11.81	(1.03)	0.11	(0.01)	1.55	(0.90)	105.6	(1.2)	187.5	(3.5)	18	(2.4)
**AB**	**I**	**Natural**	14.17	(0.98)	0.13	(0.009)	2.93	(0.85)	101.7	(1.2)	191.7	(3.4)	35.5	(2.3)
**AB**	**I**	**Orchard**	15.9	(1.03)	0.11	(0.009)	3.87	(0.90)	104.6	(1.2)	196.3	(3.5)	32.1	(2.3)
**BC**	**BV low**	**Natural**	11.21	(0.96)	0.12	(0.009)	1.81	(0.84)	105.4	(1.2)	177.9	(3.3)	34.5	(2.3)
**BC**	**BV low**	**Orchard**	20.88	(0.76)	0.16	(0.007)	7.15	(0.65)	103.4	(0.9)	207.2	(2.6)	41.1	(2.1)
**BC**	**EK all**	**Natural**	21.35	(0.91)	0.15	(0.008)	7.02	(0.79)	97.4	(1.1)	203.2	(3.1)	50	(2.2)
**BC**	**EK all**	**Orchard**	28.31	(1.03)	0.19	(0.009)	15.84	(0.90)	96.9	(1.2)	206.8	(3.5)	42.7	(2.3)
**BC**	**NE low**	**Natural**	23.08	(1.12)	0.16	(0.01)	8.31	(0.97)	94.4	(1.4)	208.8	(3.8)	58.5	(2.4)
**BC**	**NE low**	**Orchard**	30.94	(1.05)	0.18	(0.009)	17.45	(0.91)	97	(1.3)	224.2	(3.6)	52.7	(2.4)
**BC**	**NE mid**	**Natural**	12.73	(0.85)	0.13	(0.008)	2.74	(0.73)	102.3	(1.0)	183.7	(2.9)	46.5	(2.2)
**BC**	**NE mid**	**Orchard**	20.53	(1.04)	0.17	(0.01)	7.97	(0.90)	99.5	(1.3)	198.9	(3.5)	51.8	(2.4)
**BC**	**PG high**	**Natural**	10.29	(1.08)	0.1	(0.011)	1.63	(0.93)	106.4	(1.3)	176.6	(3.6)	39	(2.4)
**BC**	**PG high**	**Orchard**	15.43	(1.06)	0.11	(0.01)	3.67	(0.92)	106	(1.3)	189.6	(3.6)	37.4	(2.4)
**BC**	**PG low**	**Natural**	14.81	(0.71)	0.13	(0.007)	3.88	(0.61)	104.5	(0.9)	185.4	(2.4)	38.9	(2.0)
**BC**	**PG low**	**Orchard**	18.89	(0.75)	0.14	(0.007)	5.39	(0.65)	101	(0.9)	202.3	(2.6)	44.4	(2.1)
**BC**	**PR mid**	**Natural**	10.7	(0.92)	0.13	(0.009)	1.96	(0.80)	104.2	(1.1)	169.5	(3.1)	24.9	(2.2)
**BC**	**PR mid**	**Orchard**	17.34	(1.04)	0.12	(0.01)	4.56	(0.90)	103.3	(1.3)	199	(3.5)	30	(2.4)
**BC**	**TO low**	**Natural**	15.8	(0.94)	0.14	(0.009)	5.03	(0.81)	103.9	(1.1)	188	(3.2)	43.9	(2.2)
**BC**	**TO low**	**Orchard**	23.92	(0.75)	0.17	(0.007)	9.16	(0.64)	98.8	(0.9)	206	(2.6)	49.5	(2.1)

Table S5. r^2 values for clines in sixteen climatic variables of the six traits and two seedling types p-values (in brackets) are statistically significant at an adjusted $\alpha = 0.0031$ cut-off value.

Climatic Variable	Seedling Height		Growth Rate		Shoot Dry Mass		Bud Break		Bud Set		Cold Injury	
	Natural	Selected	Natural	Selected	Natural	Selected	Natural	Selected	Natural	Selected	Natural	Selected
**LAT**	0.42<br>(0.012)	**0.75**<br>(0.0001)	0.43<br>(0.01)	**0.77**<br>(< 0.0001)	0.45<br>(0.009)	**0.78**<br>(< 0.0001)	0.37<br>(0.02)	**0.66**<br>(0.0005)	0.15<br>(0.167)	0.43<br>(0.011)	**0.86**<br>(< 0.0001)	**0.85**<br>(< 0.0001)
**LONG**	0.05<br>(0.444)	0.03<br>(0.531)	0.02<br>(0.591)	0.02<br>(0.656)	0.04<br>(0.497)	0.03<br>(0.58)	0.06<br>(0.387)	0.00<br>(0.89)	0.37<br>(0.022)	0.12<br>(0.218)	0.04<br>(0.486)	0.01<br>(0.714)
**ELEV**	0.00<br>(0.98)	0.07<br>(0.362)	0.07<br>(0.379)	0.15<br>(0.171)	0.00<br>(0.884)	0.12<br>(0.217)	0.00<br>(0.921)	0.17<br>(0.149)	0.08<br>(0.318)	0.01<br>(0.772)	0.30<br>(0.043)	0.23<br>(0.079)
**MAT**	**0.58**<br>(0.0015)	**0.68**<br>(0.0003)	**0.61**<br>(0.001)	**0.59**<br>(0.001)	**0.64**<br>(0.0006)	**0.69**<br>(0.0002)	**0.56**<br>(0.002)	**0.59**<br>(0.001)	0.21<br>(0.096)	**0.54**<br>(0.003)	**0.91**<br>(< 0.0001)	**0.87**<br>(< 0.0001)
**MWMT**	0.24<br>(0.075)	0.13<br>(0.21)	0.04<br>(0.486)	0.07<br>(0.37)	0.21<br>(0.1)	0.08<br>(0.32)	0.27<br>(0.057)	0.08<br>(0.32)	**0.51**<br>(0.0043)	0.29<br>(0.047)	0.00<br>(0.99)	0.01<br>(0.6814)
**MCMT**	0.26<br>(0.061)	0.55<br>(0.0023)	0.45<br>(0.0082)	**0.54**<br>(0.0029)	0.31<br>(0.039)	**0.60**<br>(0.0012)	0.26<br>(0.063)	**0.53**<br>(0.0031)	0.01<br>(0.68)	0.29<br>(0.046)	**0.82**<br>(< 0.0001)	**0.90**<br>(< 0.0001)
**log MAP**	0.15<br>(0.18)	0.03<br>(0.58)	0.19<br>(0.16)	0.04<br>(0.51)	0.18<br>(0.13)	0.04<br>(0.48)	0.25<br>(0.068)	0.06<br>(0.41)	0.01<br>(0.75)	0.01<br>(0.76)	**0.69**<br>(0.0002)	0.41<br>(0.014)
**MSP**	0.03<br>(0.55)	0.21<br>(0.01)	0.05<br>(0.42)	0.27<br>(0.056)	0.01<br>(0.717)	0.17<br>(0.146)	0.00<br>(0.98)	0.19<br>(0.11)	0.00<br>(0.89)	0.04<br>(0.506)	0.01<br>(0.807)	0.11<br>(0.258)
**SHM**	0.13<br>(0.2)	0.44<br>(0.009)	0.12<br>(0.22)	0.45<br>(0.009)	0.09<br>(0.308)	0.36<br>(0.025)	0.05<br>(0.456)	0.38<br>(0.019)	0.09<br>(0.29)	0.21<br>(0.098)	0.02<br>(0.615)	0.19<br>(0.117)
**DD < 0**	0.32<br>(0.035)	**0.57**<br>(0.0017)	0.46<br>(0.0073)	**0.53**<br>(0.003)	0.37<br>(0.0202)	**0.62**<br>(0.0008)	0.30<br>(0.044)	0.51<br>(0.004)	0.04<br>(0.497)	0.35<br>(0.025)	**0.85**<br>(< 0.0001)	**0.90**<br>(< 0.0001)
**DD > 5**	0.46<br>(0.008)	0.29<br>(0.045)	0.16<br>(0.153)	0.18<br>(0.13)	0.44<br>(0.01)	0.23<br>(0.082)	0.49<br>(0.005)	0.21<br>(0.096)	**0.65**<br>(0.0005)	0.46<br>(0.007)	0.07<br>(0.356)	0.13<br>(0.206)
**EFFP**	0.39<br>(0.017)	0.37<br>(0.0206)	0.39<br>(0.0174)	0.37<br>(0.021)	0.43<br>(0.0113)	0.34<br>(0.028)	0.52<br>(0.0035)	0.39<br>(0.0173)	0.21<br>(0.1002)	0.38<br>(0.0193)	**0.64**<br>(0.0006)	**0.64**<br>(0.0006)
**PAS**	0.05<br>(0.44)	0.02<br>(0.66)	0.11<br>(0.247)	0.06<br>(0.404)	0.10<br>(0.269)	0.01<br>(0.72)	0.10<br>(0.26)	0.06<br>(0.385)	0.01<br>(0.76)	0.02<br>(0.66)	0.56<br>(0.002)	0.32<br>(0.034)
**EMT**	0.36<br>(0.024)	**0.63**<br>(0.0007)	0.38<br>(0.02)	**0.64**<br>(0.0006)	0.37<br>(0.02)	**0.61**<br>(0.0009)	0.38<br>(0.019)	**0.59**<br>(0.001)	0.09<br>(0.29)	0.41<br>(0.014)	**0.87**<br>(< 0.0001)	**0.94**<br>(< 0.0001)
**EXT**	0.31<br>(0.037)	0.34<br>(0.028)	0.07<br>(0.346)	0.28<br>(0.053)	0.26<br>(0.063)	0.25<br>(0.066)	0.27<br>(0.057)	0.27<br>(0.055)	0.52<br>(0.0037)	0.38<br>(0.019)	0.02<br>(0.669)	0.11<br>(0.24)
**CMD**	0.30<br>(0.04)	**0.62**<br>(0.0009)	0.21<br>(0.1003)	**0.59**<br>(0.0014)	0.24<br>(0.076)	0.53<br>(0.0032)	0.13<br>(0.2)	0.50<br>(0.0048)	0.19<br>(0.116)	0.35<br>(0.026)	0.12<br>(0.23)	0.34<br>(0.028)
**PC1**	**0.70**<br>(0.0002)	**0.72**<br>(< 0.0001)	0.56<br>(0.002)	**0.64**<br>(0.0006)	**0.71**<br>(0.0002)	**0.67**<br>(0.0003)	**0.68**<br>(0.0003)	**0.64**<br>(0.0006)	0.43<br>(0.011)	**0.60**<br>(0.001)	**0.71**<br>(< 0.0001)	**0.71**<br>(< 0.0001)
**PC2**	0.00<br>(0.91)	0.02<br>(0.66)	0.08<br>(0.33)	0.04<br>(0.47)	0.01<br>(0.79)	0.04<br>(0.476)	0.00<br>(0.83)	0.04<br>(0.49)	0.11<br>(0.24)	0.01<br>(0.7)	0.38<br>(0.019)	0.27<br>(0.059)

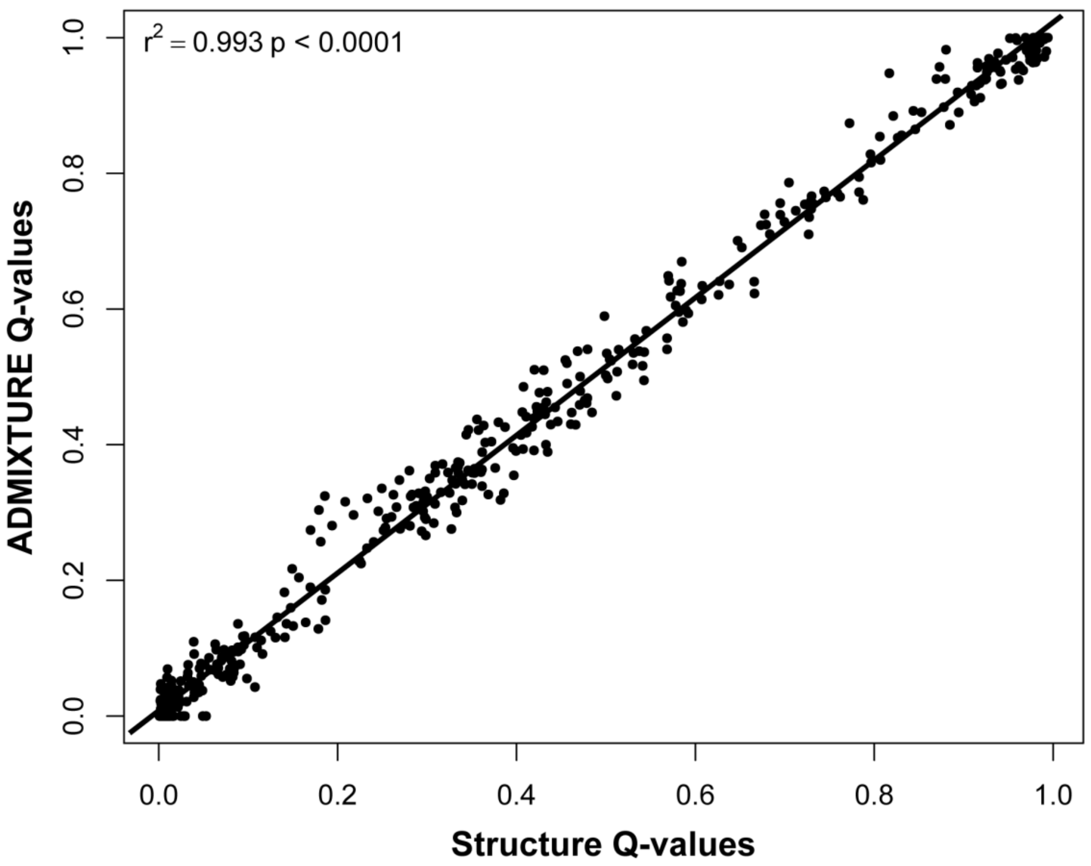

Figure S1. Comparison of ADMIXTURE and *Structure* Q-values for 500 seedlings used in the ADMIXTURE projection analysis reference panel. ADMIXTURE Q-values are estimated from 6482 neutral or candidate adaptive SNPs. Structure Q-values are estimated from 817 neutral SNPs.

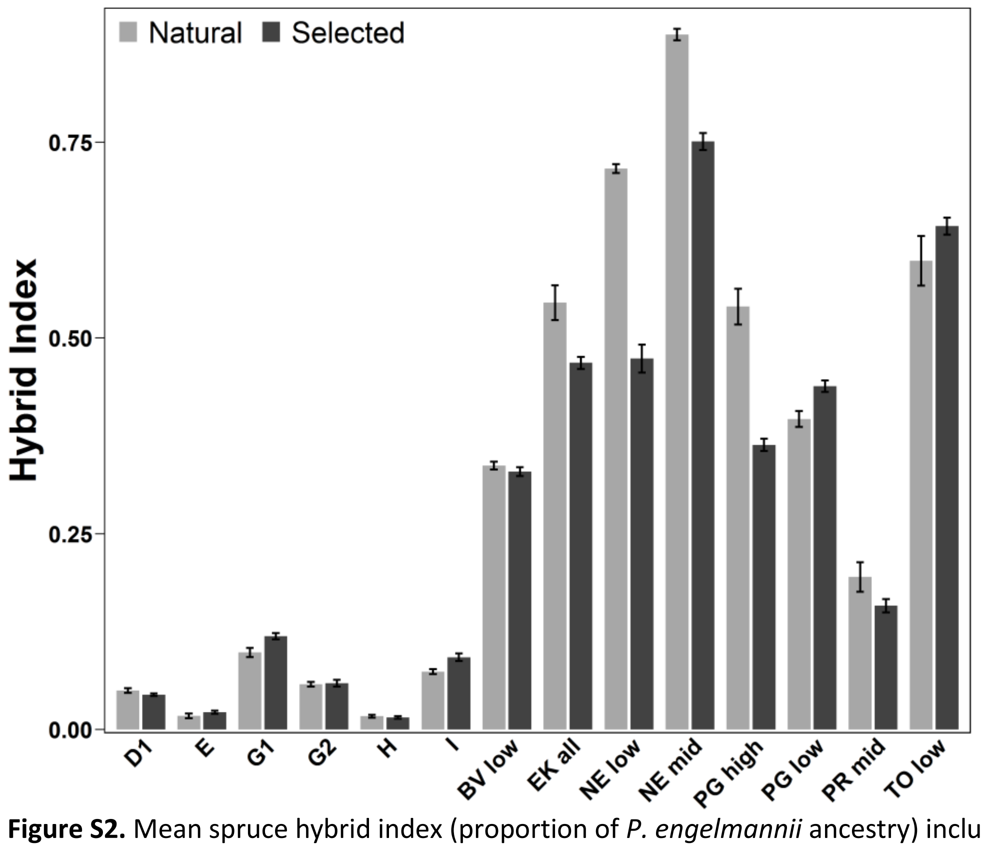

Figure S2. Mean spruce hybrid index (proportion of *P. engelmannii* ancestry) including standard errors, for each breeding zone by seedling type combination.

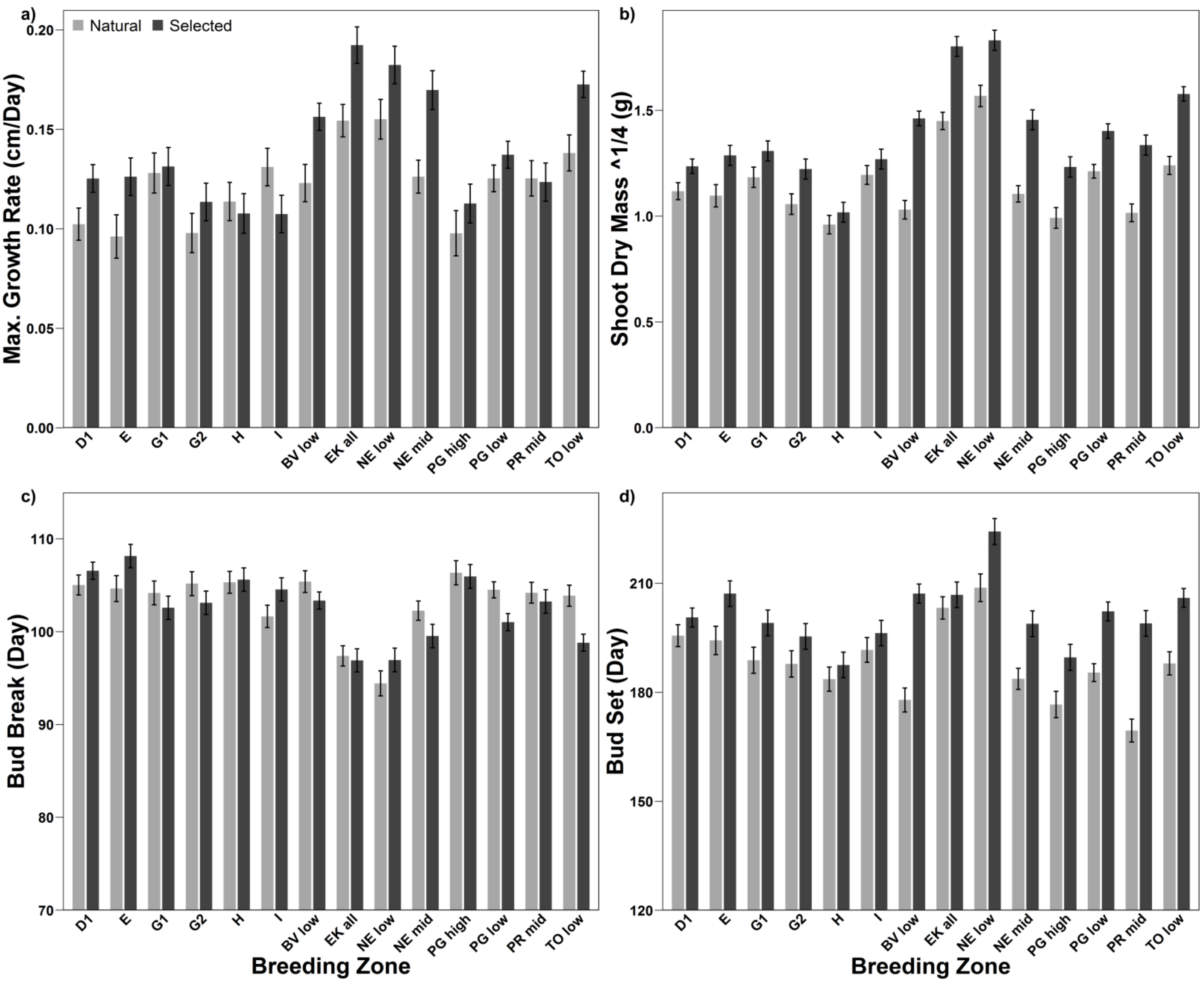

Figure S3. Bar plots of breeding zone level trait means (BLUEs) including standard error bars for a) growth rate, b) shoot dry mass, c) bud break, and d) bud set.

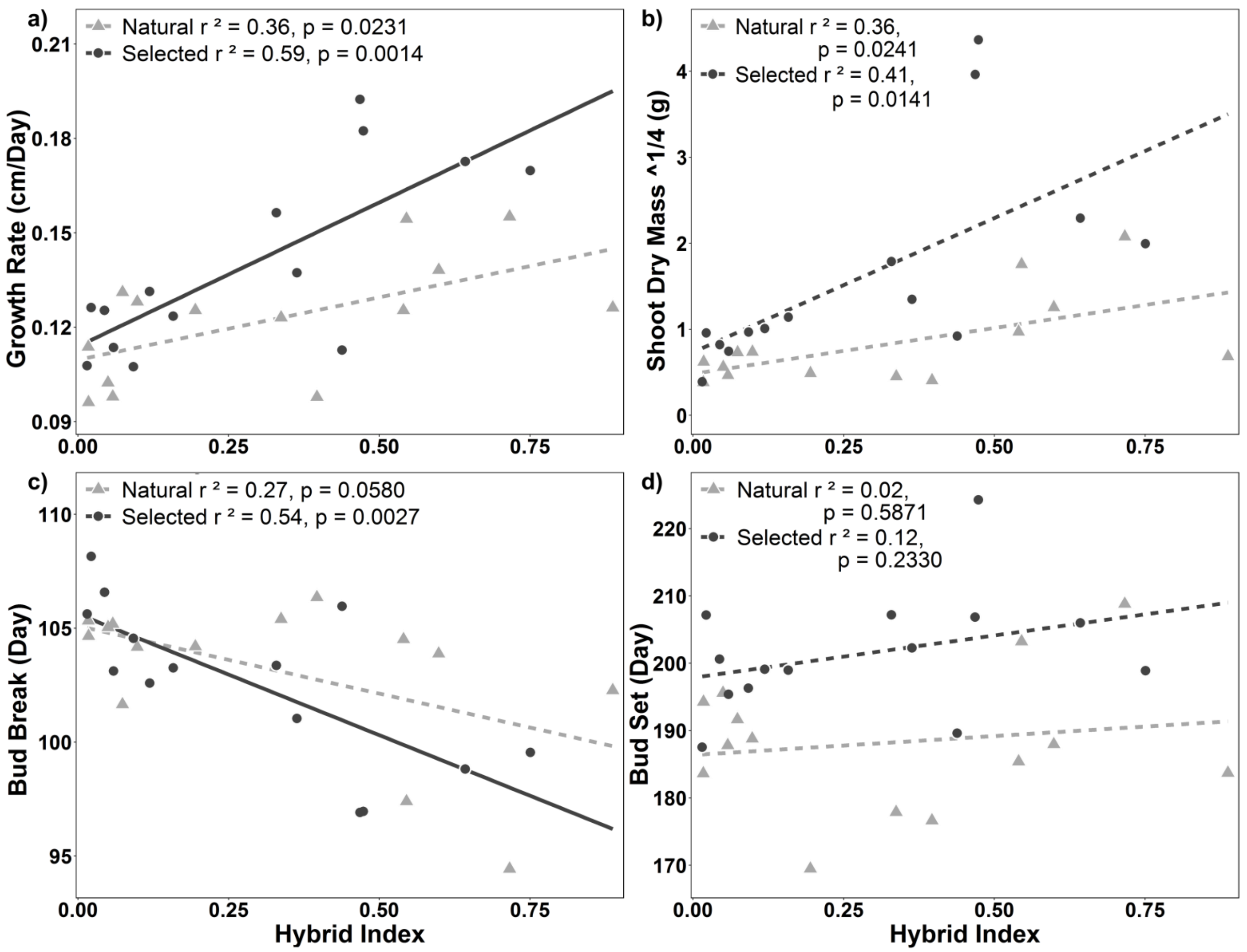

Figure S4. Complimenting figure 5, regressions of a) growth rate, b) shoot mass, c) bud break, and d) bud set, versus hybrid index. Points represent BLUEs of trait means (Table S5), and the mean spruce hybrid index (*P. engelmannii* proportion) for each breeding zone by seedling type combination. p-values are statistically significant at the adjusted α = 0.0083 cut-off used in Table 3.

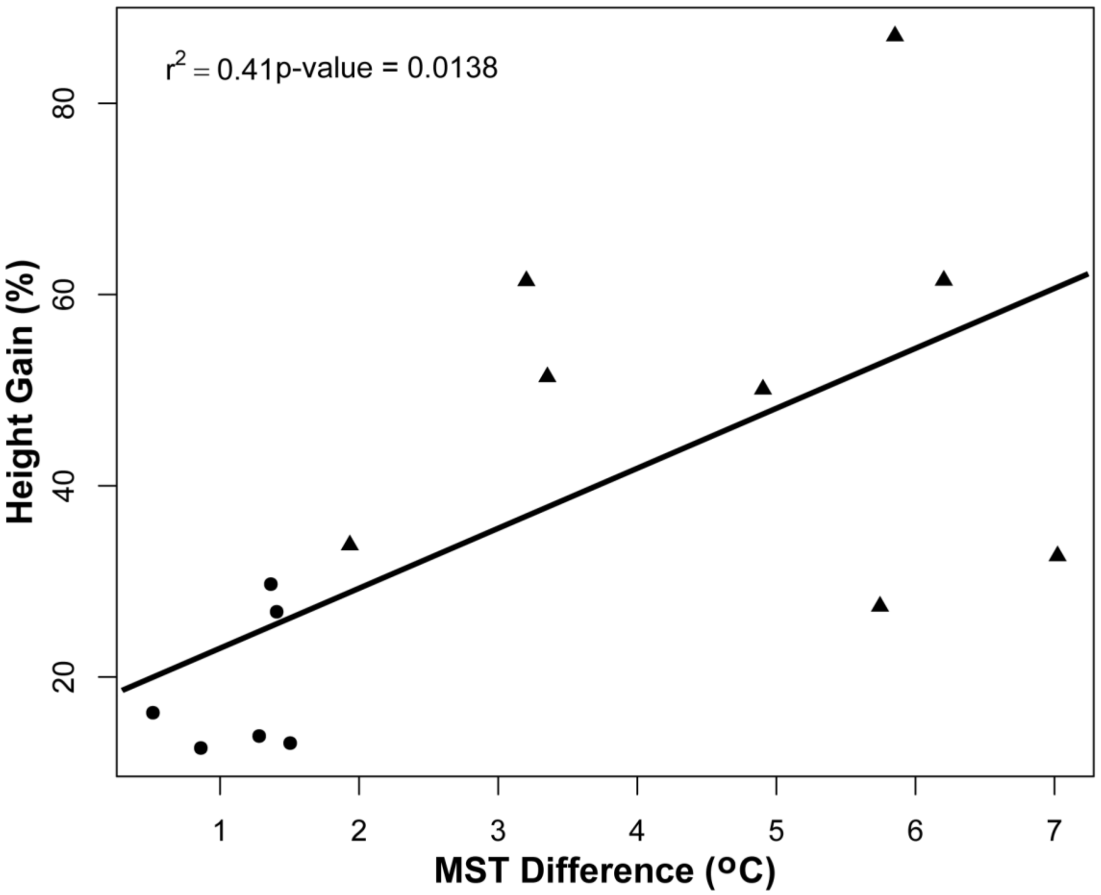

Figure S5. Regression of height gains in each breeding zone upon differences between the mean summer temperatures (June to August) of breeding zones for selected seedlings and their respective seed orchard sites.
